# Building a transnational biodiversity geo-database of the protected areas in the Adriatic-Ionian Macro-Region: approaches and results from the IMPRECO Project

**DOI:** 10.3897/BDJ.9.e67169

**Published:** 2021-05-27

**Authors:** Francesco Zangaro, Gabriele Marini, Valeria Specchia, Matteo De Luca, Francesca Visintin, Giovanna Bullo, Jacopo Richard, Nataša Šalaja, Bia Rakar, Bojana Lipej, Jelena Kurtović Mrčelić, Gvido Piasevoli, Ante Žuljević, Nada Zaimi, Djana Bejko, Abdulla Diku, Aliki Karousou, Eleni Hatziyanni, Massimiliano Pinat, Maurizio Pinna

**Affiliations:** 1 Department of Biological and Environmental Sciences and Technologies, DiSTeBA, University of Salento, S.P. Lecce-Monteroni, 73100, Lecce, Italy Department of Biological and Environmental Sciences and Technologies, DiSTeBA, University of Salento, S.P. Lecce-Monteroni, 73100 Lecce Italy; 2 Research Centre for Fisheries and Aquaculture of Aquatina di Frigole, DiSTeBA, University of Salento, 73100, Lecce, Italy Research Centre for Fisheries and Aquaculture of Aquatina di Frigole, DiSTeBA, University of Salento, 73100 Lecce Italy; 3 Nature Reserve of Isonzo Rivermouth, For-Nature S.r.l, Via T. Ciconi 26, 33100, Udine, Italy Nature Reserve of Isonzo Rivermouth, For-Nature S.r.l, Via T. Ciconi 26, 33100 Udine Italy; 4 Friuli Innovazione Research and Technology Transfer Centre, Via Linussio 51, 33100, Udine, Italy Friuli Innovazione Research and Technology Transfer Centre, Via Linussio 51, 33100 Udine Italy; 5 Veneto Agricoltura, Veneto Region's Agency for the innovation in the primary sector, Viale dell’Università 14, 35020, Legnaro, Italy Veneto Agricoltura, Veneto Region's Agency for the innovation in the primary sector, Viale dell’Università 14, 35020 Legnaro Italy; 6 DOPPS-BirdLife Slovenia, Tržaška cesta 2, 1000, Ljubljana, Slovenia DOPPS-BirdLife Slovenia, Tržaška cesta 2, 1000 Ljubljana Slovenia; 7 Public Institution for the Management of Protected Areas in the County of Split and Dalmatia “Sea and Karst”, Prilaz braće Kaliterna 10, 21000, Split, Croatia Public Institution for the Management of Protected Areas in the County of Split and Dalmatia “Sea and Karst”, Prilaz braće Kaliterna 10, 21000 Split Croatia; 8 Institute of Oceanography and Fisheries, Laboratory for benthos, Split, Croatia Institute of Oceanography and Fisheries, Laboratory for benthos Split Croatia; 9 Albanian Development Fund, Sami Frasheri 10, 1000, Tirana, Albania Albanian Development Fund, Sami Frasheri 10, 1000 Tirana Albania; 10 Region of Crete, Directorate of Environment and Spatial Planning, Eleftherias Street, 71201, Heraklion, Greece Region of Crete, Directorate of Environment and Spatial Planning, Eleftherias Street, 71201 Heraklion Greece; 11 Institute for Marine Biological Resources and Biotechnology of the National Research Council (CNR-IRBIM), Via S. Raineri 86, 98122, Messina, Italy Institute for Marine Biological Resources and Biotechnology of the National Research Council (CNR-IRBIM), Via S. Raineri 86, 98122 Messina Italy

**Keywords:** Coastal-Marine Protected Areas, European Adriatic-Ionian Macro-Region, EUSAIR, NATURA 2000, non-indigenous species, protected species and habitats, traditional and innovative biomonitoring tools, transnational biodiversity geo-database.

## Abstract

**Background:**

The main objective of the project *Common strategies and best practices to IMprove the transnational PRotection of ECOsystem integrity and services* - IMPRECO is to enhance the safeguarding of ecosystems and ecosystem services. Additionally, the aim of this project is to tackle their environmental vulnerability by strengthening the potential of the Protected Areas in biodiversity, ecosystems and ecosystem services conservation. This is expected to be addressed by maintaining it through their transnational networking located in the European Adriatic-Ionian Macro-Region.

**New information:**

The aim of this research is: 1) to characterise the habitats and ecosystems involved in the coastal-marine protected areas considered; 2) to set a biodiversity baseline; 3) to understand what current ecosystems’ conditions are; 4) to build up a transnational biomonitoring programme of target species and habitats and 5) to assess their response to pilot actions. To do so, a transnational inventory of species, habitats, ecosystems and ecosystem services was established, starting with the seven coastal-marine protected areas involved in the project. Data collection was carried out using different sources of information: scientific literature, officially available data from NATURA 2000 Standard Data Forms, checklists from local biomonitoring programmes, personal observations and citizen science, historical maps and data from new in-field analyses. Data were filled in the transnational biodiversity geo-databases according to the NATURA 2000 standards about habitat features, species protection level and species features. The presence of alien species (non-indigenous species, NIS) was also acknowledged and references about data collection were provided in the databases according to the Darwin Core standards.

## Introduction

Since the 2000s, a globally-shared challenge is to maintain the supply of ecosystem services - commonly defined as the benefits to humans deriving from the ecosystems’ dynamics - without impacting the biodiversity, processes and quality of an ecosystem (Birk et al. 2012, Lin and Egerer 2020, Pinna et al. 2016, Russo et al. 2018). In order to protect ecosystems from damage and to restore degraded ones to a “good status”, extensive regional, national, international and transnational regulations have been adopted. However, the application of transnational approaches is still not largely diffused, resulting in an innovative collaborative working activity. A crucial prerequisite to these regulations is to conduct accurate assessments for every environmental protection and recovery programme (Birk et al. 2012, Pawlowski et al. 2018, Specchia et al. 2020). From the 2000s onwards, the ecological status of ecosystems has been monitored mainly by characterising the biological communities, functions and processes (Fonnesu et al. 2004, Pinna et al. 2003, Pinna et al. 2017, Sangiorgio et al. 2014), because the occurrence and the abundance of the species, considered as good biological and ecological indicators, are known to be essential in determining the ecological status of the different ecosystems (Birk et al. 2012, Borja et al. 2013, Di Sabatino et al. 2014). To address this, a large number of biotic indices has been developed in many different countries, based on the morphological identification of various organisms at a different level of organisation (Birk et al. 2012, Borja et al. 2013, Di Sabatino et al. 2014). Innovative DNA-based tools have also been developed to support the monitoring of biodiversity of aquatic ecosystems (Tzafesta et al. 2021).

*Common strategies and best practices to IMprove the transnational PRotection of ECOsystem integrity and services* – IMPRECO is a territorial cooperation project, funded by the first call of the INTERREG VB ADRION 2014-2020 Programme, Priority Axis 2 “Sustainable Region” (IMPRECO 2018). The purpose of this project is: 1) to set a biodiversity baseline of the ecosystems involved in order to understand what the current ecosystems’ conditions are; 2) to build up a transnational biomonitoring programme of target species and habitats; and 3) to assess their response to different pilot actions.

The IMPRECO project involves seven project partners, including the Municipality of Staranzano, Veneto Agricoltura, University of Salento, DOPPS–BirdLife Slovenia, “Sea and Karts”, Albanian Development Fund and the Region of Crete. The seven project partners presented seven protected areas including Foce dell’Isonzo Nature Reserve, Bosco Nordio Integral Reserve, Aquatina di Frigole, Škocjanski zatok Nature Reserve, Pakleni Islands, Shkodra Lake and Buna Delta, North-eastern Peninsula of Crete (IMPRECO 2018).

The seven protected areas are highly heterogeneous in terms of habitat types (Galuppo et al. 2007), present animal and plant communities and protection and threatened levels, thus requiring different protection strategies (IMPRECO 2018).

### Study area description

**Figure 1.**
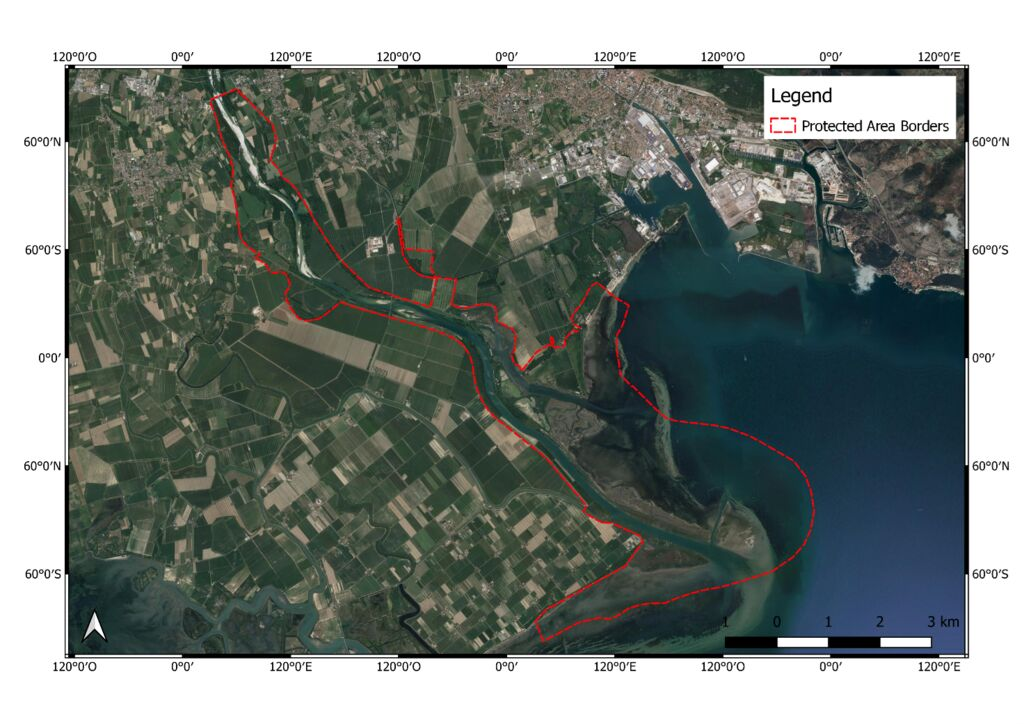
Foce dell’Isonzo – Isola della Cona (IT3330005) Protected Area.

**Foce dell’Isonzo – Isola della Cona Protected Area** (IT3330005; Fig. 1) is located in the eastern part of the Friuli-Venezia Giulia Region (Northern Italy), along the final section of the Isonzo River. It covers an area of 2,338 hectares, 1,154 ha of which are marine environments. The Reserve stretches up to the far east of the Po Valley (Isonzo Plain) and includes the final part of the fluvial section on the high plain. It is characterised by evident gravelly alluvium, while in areas on the low plain, with their predominantly loamy soils, we find extensive reclamation areas from the twentieth century. In this section, there are still several remnants of an alluvial forest and channelled spring watercourses. In the most southern part of this area, we find an entire estuary environment, most of which is still marshland, featuring brackish and salt clayey alluvium (flood plains, sandbanks and mudflats), as well as sand deposits on the bar of the estuary, which emerge as a few small islands. In this area, over 330 species of migratory, winter, summer and non-migratory birds can be observed. The main source of threat for this area appears to be the presence of invasive alien species, threatening human health and biodiversity and with socio-economic consequences too (IMPRECO 2018).

**Figure 2.**
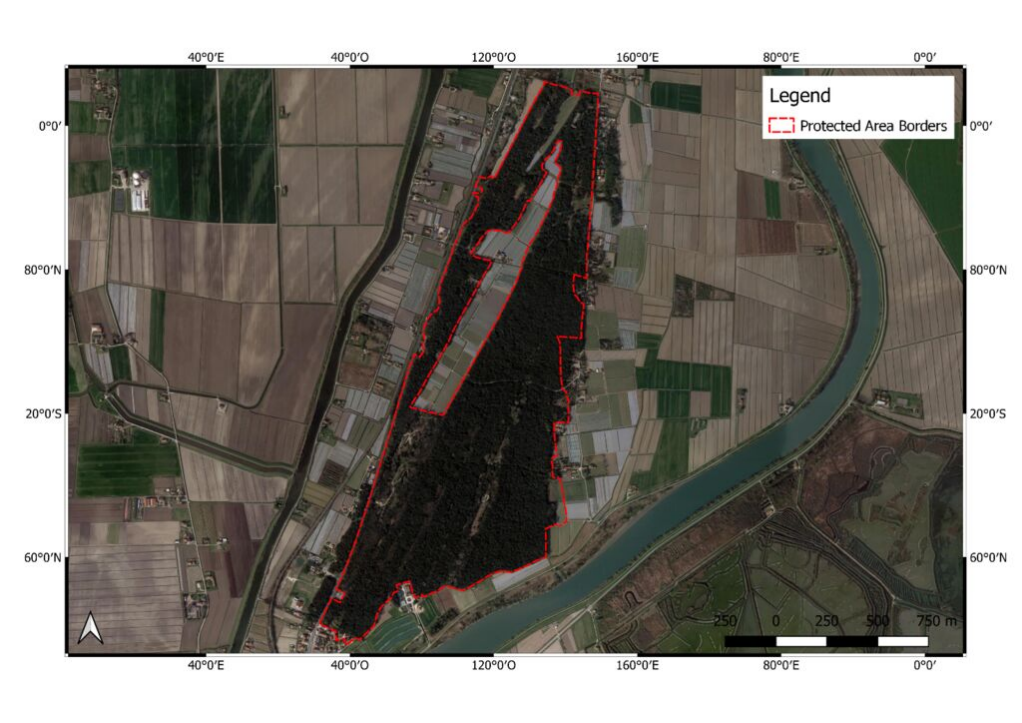
Bosco Nordio (IT3250032) Protected Area.

**Bosco Nordio Protected Area** (IT3250032; Fig. 2) is situated on the most ancient dune system of the coastal area to the south of Venice (Veneto Region, northern Italy), which is the result of the building activity done by the rivers Po and Adige during the last 4,000 years. Bosco Nordio seems to have pre-Roman origins. Today, only about 160 hectares of the original forest remain. In 1959, 113 hectares were sold to the State and became a national Nature Reserve with decree D.M. 26/7/1971. *Quercus ilex*, *Quercus robur* and *Fraxinus ornus* are the most common tree species. The clearings in the forest show rare, typical vegetation of the ancient dunes (“Grey dunes”). Bosco Nordio presents eight habitats of Community interest (three are priority habitats). The Reserve has important herpetofauna, with a total of 20 species. The surrounding area shows widespread urbanisation, which fragments an agricultural territory in which the original natural environments are reduced to parcels often of minute dimensions. In this context, the most sensitive species (for example, the Common Viper - *Vipera aspis*) have now disappeared and the surviving ones are directly threatened by the extension of the road network, which further fragments the already-reduced territories occupied by these animals and which can decimate in a short time their populations. More generally, in this area reptiles suffer from the simplification of the territory for agricultural purposes, with the elimination of those natural elements that represent shelter for many species (for example, the natural bands around the ditches for the European pond turtle *Emys orbicularis* or the bushy meadows on the banks of the rivers for the Green Lizard *Lacerta bilineata*; IMPRECO 2018).

**Figure 3.**
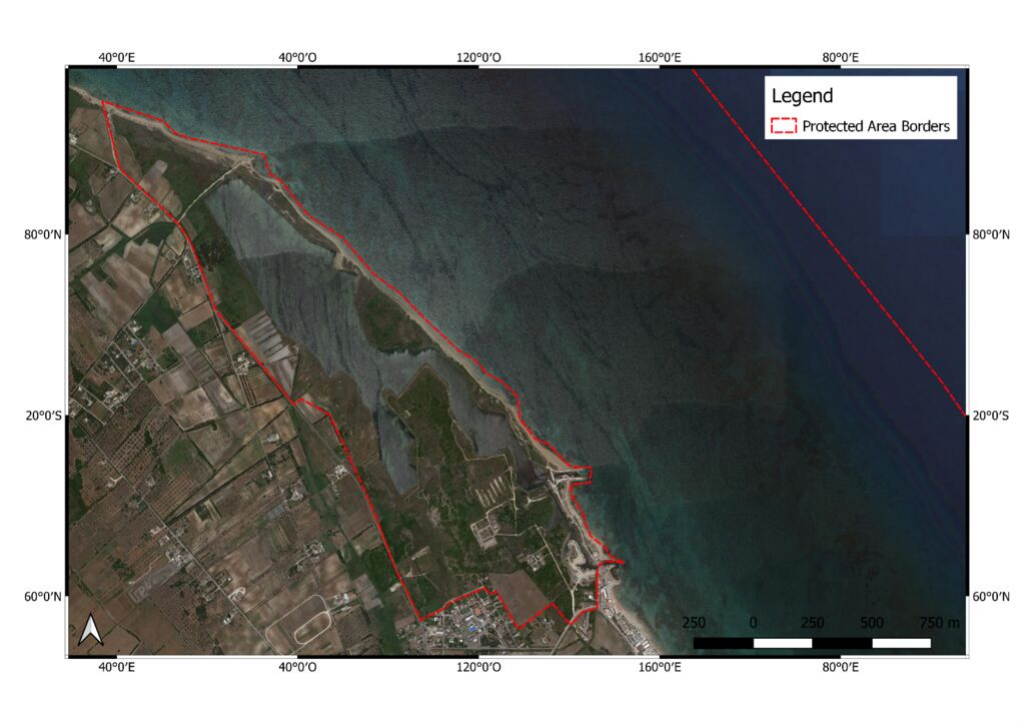
Aquatina di Frigole (IT9150003) Protected Area.

**Aquatina di Frigole Protected Area** (IT9150003; Fig. 3) is located on the Adriatic Sea coastline of the Salento Peninsula (Apulia Region, south-eastern Italian coast, 40.4425°N, 18.2376°E), about 13 km northeast of the town of Lecce (Italy). The NATURA 2000 site covers both coastal-marine and terrestrial habitats. A coastal lagoon of about 43 hectares is also included in the NATURA 2000 site. Information concerning both the environment ecological quality and the fisheries inside the lagoon was already available in 18^th^ century publications, but the first scientific paper on the fish yield was published when the lagoon was proposed to develop aquaculture activities in 1982 (Rossi and Corbari 1982). The Aquatina di Frigole NATURA 2000 site is characterised by a high variety of fauna and flora species, which can be divided into the following groups: plants, algae, zooplankton, macroinvertebrates assemblages, fishes, amphibians, reptiles, birds and mammals. Recently, the presence of Mediterranean endemic species (e.g. *Pinna nobilis* and *Monachus monachus*) has been acknowledged for the first time in the Protected Area (Marrocco et al. 2018, Zangaro et al. 2020). In addition, threatening factors for this area, such as alien species (e.g. *Callinectes sapidus*) have been recognised, as well as the impacts due to the diffusion of plastics and micro-plastics in the area and on the nearby shoreline have been underlined (Frigione et al. 2021, IMPRECO 2018).

**Figure 4.**
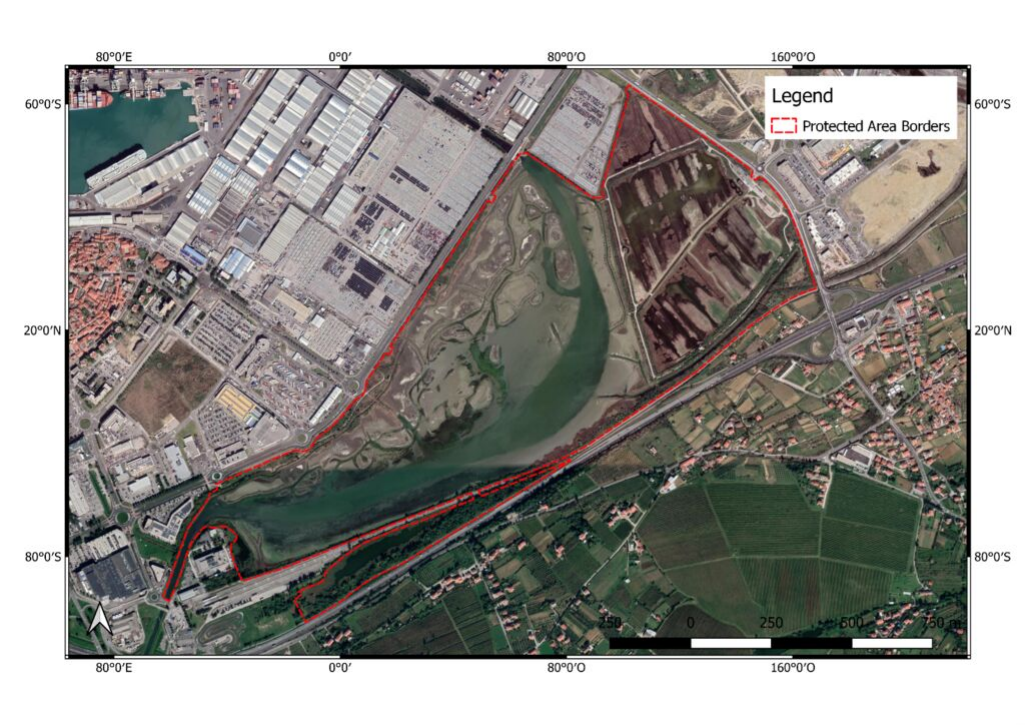
Škocjanski zatok (SI3000252) Protected Area.

**Škocjanski zatok Protected Area** (SI3000252; Fig. 4) is a coastal wetland located in the outskirts of the city of Koper on the Slovenian coast in the north-eastern Adriatic. The Reserve hosts a great diversity of birds and other animal and plant species. Birds are the most outstanding fauna group: the Reserve area of only 122.7 hectares hosts over 259 species and the number of observed birds is still increasing. This number is greater than 60% of all the bird species observed in Slovenia. The construction of the facilities in this area followed the principles of sustainable building. The area shows a wide range of plant and animal life, including many rare and endangered species. Camargue horses and Podolian cattle, which help to maintain the vegetation balance of the freshwater marsh, add to the diversity of the Reserve. Due to the strong urbanisation around this area, it is threatened by a strong human pressure, mainly derived from light pollution, water pollution and noise. Additionally, the the presence of invasive alien species has been reported in this area (IMPRECO 2018).

**Figure 5.**
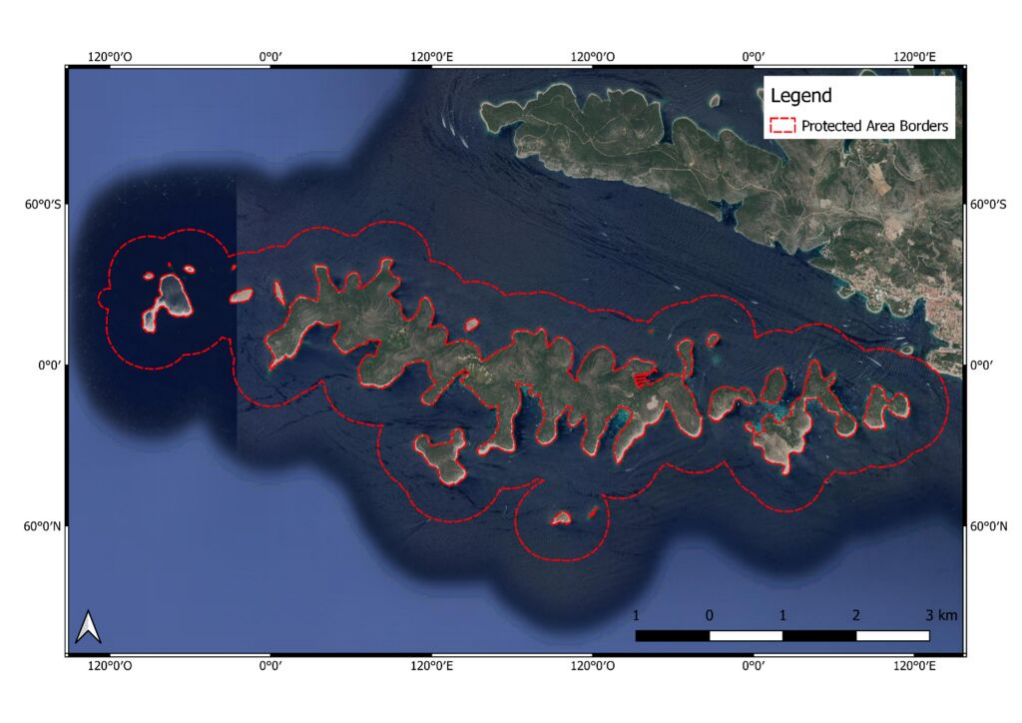
Pakleni Otoci Islands (HR3000095) Protected Area.

**Pakleni Otoci Islands Protected Area** (HR3000095; Fig. 5) is an archipelago of 19 islands and islets, belonging to over 1000 islands belonging to Croatia. They show a complex and deeply indented geography, stretching over 634.38 ha. The undersea environments surrounding the islands are part of the European Ecological NATURA 2000 Network which has the aim of preserving meadows of *Posidonia oceanica*, reef habitat with established infralittoral algal and coralligenous communities, infralittoral sandy bottoms and marine caves. The islands are made of limestone and, on the largest, Sv. Clement, the dry land reaches a height of 96 m. Vinogradišce Bay is remarkable for its diluvia sand which forms a picturesque sandy beach. The natural vegetation is made up primarily of machete and Aleppo pine that, with their limestone shorelines, give the islands their characteristic green, bordered with a white appearance from the air. The biodiversity of the islands’ marine habitats is characterised by the western region of the Archipelago, which includes the large island of Vodnjak with its surrounding islets and reefs, Močiguzica Point and the Island of Stambedar with its Pločica islets. Every island and islets are almost completely surrounded by a dense, well developed meadow of *Posidonia oceanica*, which forms a priority NATURA 2000 habitat. Other important habitats, such as coralligenous communities and sea caves, are exceptionally developed on the most southern and most western area. Amongst such exceptional micro-locations are the shallows near the Vodnjak Veliki Island, known as Kampanel. The Kampanel shoal is a complex underwater rocky ridge extending from 10 m to nearly 60 m deep. At a depth of 15 m, there is a very well-developed coral community with large and dense settlements of gorgonian species *Paramuricea clavata* and *Eunicella cavolini*, as well as numerous cracks and fissures with a complex of sea caves. The area is highly threatened mainly by anthropogenic threats (e.g. piers and marinas; municipal waste landfills and other landfills, including trash and solid waste; illegal fisheries and harvesting of aquatic resources; illegal diving activities; illegal water sports). In addition, invasive alien species have been reported to be present in this area (IMPRECO 2018).

**Figure 6.**
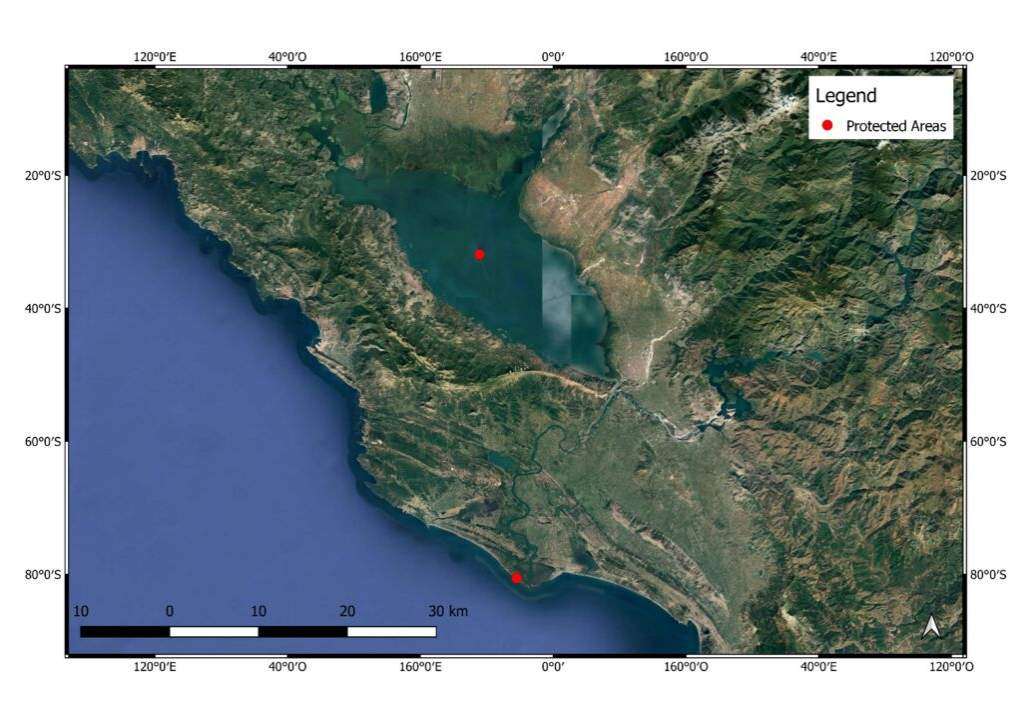
Shkodra Lake and Buna Delta Protected Areas.

**Shkodra Lake**, the largest lake on the Balkan Peninsula (Fig. 6), is located on the border between Montenegro and Albania, in the southern part of the Dinaric Alps. The catchment basin is about 5,500 km^2^ (4,470 km^2^ in Montenegro and 1,030 km^2^ in Albania) and flows south-east into the Adriatic via the Buna River. The Buna River is the outflow of the Lake Shkoder and receives the waters of the Drin River Basin with a total area of about 21,000 km^2^.

**The Buna River Protected Area** (Fig. 6) comprises one of the most important coastal wetland areas of the country. Situated around the Delta of the Buna River, the Park supports a great variety of wetland communities. Together with recent coastal dune deposits and inland low karst ridges, the landscape includes a wide diversity of geological types, landscapes, habitats and plant and animal species. Actually, the NATURA 2000 network is not applied in Albania. However, Albania has a National Regulation for the protection of species, named the “Emerald Network”. Threats for Shkodra Lake and Buna River arise from past and present practices of drainage for agriculture; uncontrolled development; changes in water regime; deforestation; illegal hunting and fishing; and introduced alien invasive species. (IMPRECO 2018).

**Figure 7.**
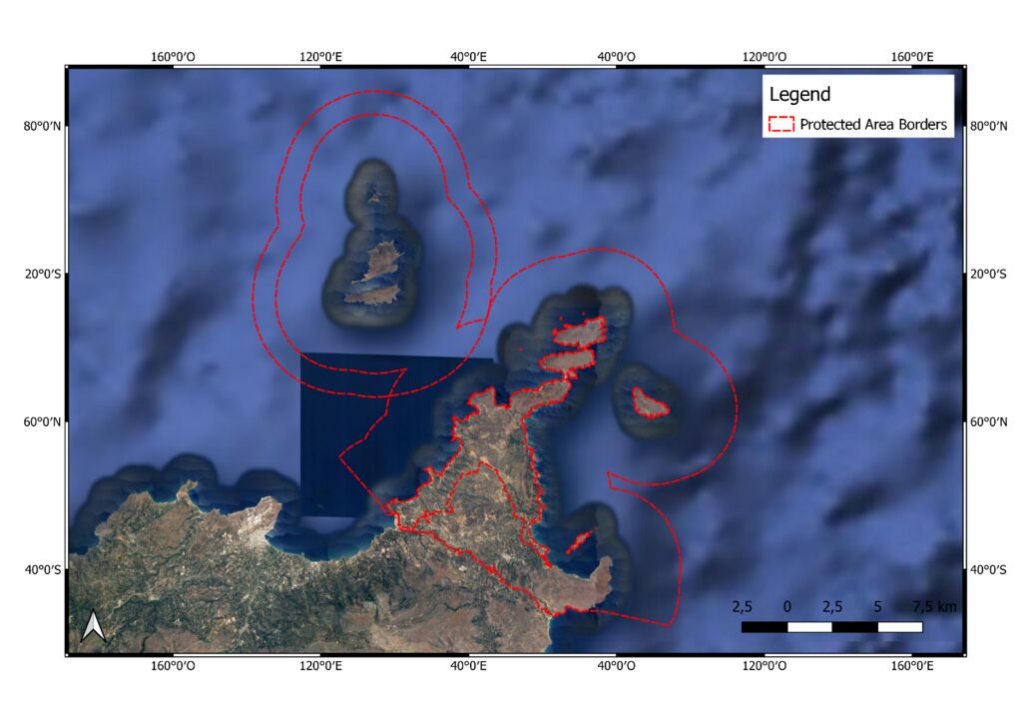
North-eastern edge of Crete (GR4320006) Protected Area.

**North-eastern Edge of Crete Protected Area** (GR4320006; Fig. 7) is composed of three partly overlapping NATURA 2000 sites. This site is a complex area of important habitats, which includes the unique in Europe Vai Palm Forest, the Sidero Peninsula, the small coastal wetlands (protected by the Greek legislation), the Dyonissades and Elassa groups of islets and their adjacent marine area. Geologically, it consists of limestones and dolomites of the upper Cretaceous, bedded crystalline limestones of the Permian, phylites and neogene and alluvial deposits. The vegetation is mainly phrygana. There are lots of valleys with maquis, some of them degrading. The Theophrastus palm forest (*Phoenix theophrastii*) of Vai is situated in a coastal valley. There are plantations of bananas in greenhouses and the land near the villages is cultivated. On the eastern coast, there are sand dunes. On the west side of Sidero Peninsula, there are *Posidonia oceanica* beds and flocks of *Tursiops truncatus* have been observed. The Dyonissades islets group consists of Permian limestone, while Elasa Islet consists of dolomites and limestones of upper Cretaceous. The vegetation on the islets is phrygana. In the larger islet of Dyonissades group, as well as on the opposite coast of Crete, the coastline is characterised by sea cliffs. In 2015, the site extended 2 nautical miles off the Cretan and surrounding islet coasts, to include important and vulnerable habitats of the circumlittoral and deep zone, including biodiversity-rich *facies* of coralligenous assemblages and extensive detritus and rhodolith beds, at depths below 40 m. Several islets, reefs and shoals add to the geographic and topographic complexity of the site. A significant number of Cretan endemic plant species and protected plant species occur in this area. The non-endemic *Lygeum spartum* belongs to the desert-like floristic element, occurring only in steppe communities in Crete, but nowhere else in Greece. Concerning fauna, a lot of invertebrate endemic species and some vertebrate endemic subspecies are present. Reptiles *Lacerta trilineata* and *Podarcis erhardii*, besides being legally protected, are also considered species of Community interest (Annex IV, Directive 92/43/EEC). The mammal *Pipistrellus savii* is considered species of Community interest (Annex IV, Directive 92/43/EEC) and protected by Greek Law (Presidential Decree 67/1981) and by the Bern Convention (Appendix II). A lot of land snail species are endemic to the site or to Crete. The Dyonissades islet group has been characterised as an Important Bird Area, especially for birds, which live at the cliffs. Species of interest are *Falco eleonorae*, *Falco naumanni* and *Calonestris diomedea*. The marine area around Dionysades and Elassa is threatened by illegal fisheries, whereas the islets themselves are vulnerable due to overgrazing. The Vai Palm Forest is threatened by underground water over-pumping for irrigation purposes and by the alien species of *Phoenix dactylifera*. Other threats are intensive cultivation, overgrazing, which is sometimes combined with intentional fire setting, illegal hunting and camping (IMPRECO 2018).

## General description

### Purpose

The aim of this research is: 1) to obtain a clear characterisation of the habitats and ecosystems involved in the project; 2) to set a biodiversity baseline; 3) to understand what the current ecosystem conditions are; 4) to build up a transnational biomonitoring programme of target species and habitats; and 5) to assess their response to pilot actions. To do so, a transnational inventory of species, habitats, ecosystems and ecosystem services has been established, starting with the seven Protected Areas that are involved in the project. Species and ecosystems inventories were established at a local level by each project partner, then merged into one large inventory. Data collection was carried out using different sources of information: scientific literature, official available data from NATURA 2000 Standard Data Forms, checklists from local biomonitoring programmes, personal observations and citizen science, historical maps and data from new in-field analyses. Data were filled in the transnational biodiversity geo-databases according to the NATURA 2000 standards about habitat features, species protection level and species features. The presence of alien species, which are rapidly increasing in the Mediterranean Sea (Bariche et al. 2020, Mannino et al. 2019), was acknowledged and references were provided, according to the Darwin Core standards.

The following data package represents the output of the data described in the Deliverable T1.1.2 - "Transnational biodiversity geo-database" of the IMPRECO project. The geo-database was approved by the project consortium and is, therefore, shared in the framework of this publication as an open access source in accordance with the requests of the European Union on the open availability of data from the projects.

## Sampling methods

### Sampling description

The pilot protected areas were selected by the project partners to be involved in the collection of biodiversity data and to compare biodiversity data amongst them on a transnational Adriatic-Ionian spatial pattern, from the northern Adriatic Sea to the north-eastern edge of the Island of Crete. The main bulk of data collection was conducted using the most recent NATURA 2000 Standard Data Forms. Nevertheless, because biodiversity is not limited only to protected species and habitats, data collection was improved by incorporating data from species checklists of local biomonitoring programmes; scientific literature retrieved from multiple databases related to cross-disciplinary research, which allow an in-depth exploration of specialised sub-fields within certain academic or scientific disciplines, such as Web of Science (http://www.webofknowledge.com), Science Direct (http://www.sciencedirect.com) and Google Scholar (http://scholar.google.com; Marrocco et al. 2019), by adding the keywords "biodiversity, species, ecosystems, ecosystem services, threatens, alien species" to the name of the Protected Areas; personal observations and observations shared through the citizen science communications and interviews; historical maps; and data obtained through new field analyses.

The resulting data were organised into two datasets (Suppl. materials 1, 2) to collect all information on species and habitats protected through the NATURA 2000 directives (Birds Directive 2009/147/EC and Habitats Directive 92/43/EEC), species protected through the Croatian Regulation on strictly protected species (OJ 80/2013, 73/2016), species protected through the Emerald Network and species and habitats that are not protected. The biodiversity templates were based on the following entries: taxonID; occurrenceID; scientificName; kingdom; phylum; class; order; family; genus; taxonRank; country; countryCode; locality; VerbatimCoordinateSystem; verbatimLatitude; verbatimLongitude; habitat; organismRemarks; establishmentMeans; bibliographicCitation; basisOfRecord, according to the Darwin Core format (Zangaro et al. 2021).

Species were listed, based on the principal taxonomic groups: Plants, Invertebrates, Birds, Fish, Algae, Mammals, Reptiles, Amphibians and Lichens and matched with the most commonly used habitats. At the same time, the presence of invasive alien species with reference to the European Alien Species Information Network – EASIN Web Site (European Commission 2020), was included. The name of the species was reviewed using international databases, such as WORMS (WORMS 2020), Avibase (Warnier 2014), AlgaeBase (Guiry 2010), EU-NOMEN (de Jong et al. 2015) and IUCN (IUCN 2020).

Data for each country and pilot project area was divided according to NATURE 2000 Directives’ species included in the Standard Data Form, listed under Birds Directive and Habitats Directive; NATURE 2000 Directives’ species included in the Standard Data Form listed under Other Interesting Species; species listed in NATURA 2000 Directives, but missing in the Standard Data Forms; and species that are not protected. Shkodra Lake and Buna Delta species, in contrast to the other Protected Areas’ species, are protected on a national level through the institution of a protection plan named "Emerald Network". Pakleni Otoci Islands species were also considered protected according tothe Croatian Regulation on strictly protected species (OJ 80/2013, 73/2016), a national level protection plan for Croatian species.

The biodiversity data were initially compiled by counting the species of the NATURE 2000 Directives by taxonomic group and habitat for each protected area. Subsequently, the observed species protected by the Habitats Directive, but missing in the standard data forms, were considered. Eventually, unprotected species observed through personal observations or found in previous checklists were included.

In the same manner, all habitat types, present in the NATURA 2000 Standard Data Forms for each Protected Area were listed with their coverage in hectares in each of the Protected Areas. The total number of habitats was reported for each Protected Area.

## Geographic coverage

### Description

European Adriatic-Ionian Macro-Region (EUSAIR)

### Coordinates

 and 40°7'43.78" N Latitude; and 18°56'52.89" E Longitude.

## Temporal coverage

**Data range:** 2018-1-01 – 2021-1-31.

## Usage licence

### Usage licence

Creative Commons Public Domain Waiver (CC-Zero)

## Data resources

### Data package title

Transnational biodiversity geo-database of the Protected Areas in the Adriatic-Ionian Macro-Region - IMPRECO Project.

### Number of data sets

2

### Data set 1.

#### Data set name

Species biodiversity transnational geo-database - IMPRECO Project

#### Number of columns

21

#### Description

Suppl. material 1

**Data set 1.** 

Column label	Column description
taxonID	An identifier for the set of taxon information.
scientificName	The full scientific name, with authorship and date of information.
kingdom	The full scientific name of the kingdom in which the taxon is classified.
class	The full scientific name of the class in which the taxon is classified.
order	The full scientific name of the order in which the taxon is classified.
family	The full scientific name of the family in which the taxon is classified.
genus	The full scientific name of the genus in which the taxon is classified.
taxonRank	The taxonomic rank of the most specific name in the scientificName.
country	The name of the country or major administrative unit in which the observation occurred.
locality	The specific description of the protected area (NATURA 2000/Emerald Network) in which the observation occurred.
verbatimLatitude	The verbatim original latitude of the Location.
verbatimLongitude	The verbatim original longitude of the Location.
habitat	A description of the habitat in which the observation occurred.
organismRemarks	A description of the protection level for the observed species.
establishmentMeans	Statement about whether or not an organism has been introduced to a given locality through the direct or indirect activity of modern humans.
bibliographicCitation	A bibliographic reference for the resource.
occurrenceID	An identifier for the Occurrence.
phylum	The full scientific name of the phylum.
countryCode	The standard code for the country in which the Location occurs.
verbatimCoordinateSystem	The name of the system in which the verbatim geographic coordinates were recorded.
basisOfRecord	The specific nature of the data record.

### Data set 2.

#### Data set name

Habitats transnational biodiversity geo-database - IMPRECO Project

#### Number of columns

9

#### Description

Suppl. material 2

**Data set 2.** 

Column label	Column description
Habitat code N2k	Code for the habitat protected by NATURA 2000.
Habitat N2k denomination	Name of the habitat protected by NATURA 2000.
Foce dell'Isonzo - Isola della Cona (ha)	Hectares covered by the habitat in the protected area.
Bosco Nordio (ha)	Hectares covered by the habitat in the protected area.
Aquatina di Frigole (ha)	Hectares covered by the habitat in the protected area.
Škocjanski zatok (ha)	Hectares covered by the habitat in the protected area.
Pakleni otoci (ha)	Hectares covered by the habitat in the protected area.
Voreioanatoliko akro kritis: dionysades, elasa kai chersonisos sidero (akra mavro mouri – vai – akra plakas) kai thalassia zoni (ha)	Hectares covered by the habitat in the protected area.
Reference	A bibliographic reference for the resource.

## Additional information

### Discussions and conclusions

The resulting data led the Project Partners to identify, as a subject for the monitoring programmes, different target species, such as *Aphanius fasciatus*, *Testudo hermanni*, *Pinna nobilis*, *Lutra lutra*, *Himantopus himantopus* and *Anacamptis pyramidalis* (IMPRECO 2018).

At the same time, it was possible to identify, for the monitoring programmes, different target habitats, such as *Posidonia oceanica* beds, *Cymodocea* and *Zostera* beds, Palm groves of *Phoenix* and Grey dunes (IMPRECO 2018).

Furthermore, the analysis of the biodiversity in the Protected Areas underlined a large gap in knowledge about the species biodiversity at a transnational Adriatic-Ionian Macro-Region spatial scale. In fact, as it is highlighted by the analysis of species protected by the NATURA 2000 Network, but not included in the Standard Data Forms, 486 species protected by the NATURA 2000 Network are present in the Protected Areas, but are not included in the Standard Data Forms. For instance, species like *Pinna nobilis* (Marrocco et al. 2018), a species that is facing the risk of extinction in these years due to mass mortality events (Marrocco et al. 2019) and *Monachus monachus*, a critically endangered species (Zangaro et al. 2020), have been observed in the Aquatina di Frigole Protected Area, underlining the importance of the monitoring programmes in the Protected Areas.

Additionally, the problem of alien invasive species has been evidenced. In fact, according to the analysis of alien species in the Protected Areas, 380 species have been identified, including highly invasive species, such as *Trachemys scripta*, *Phragmites australis*, *Callinectes sapidus* and *Myocastor coypus* (IMPRECO 2018).

Different achievements have been reached during the monitoring programme. For instance, new monitoring tools, such as the use of environmental DNA (eDNA; Specchia et al. 2020, Tzafesta et al. 2021), have been identified. In addition, as already mentioned, through the monitoring programmes different protected and endangered species never reported before have been observed in the Protected Areas (IMPRECO 2018).

In conclusion, due to the results achieved by this transnational programme, it can be assumed that the network represented a pilot action for implementing future transnational monitoring programmes in the European Adriatic-Ionian Macro-Region.

## Supplementary Material

113E1279-D7BE-5CDA-8CF0-795530C47CEE 10.3897/BDJ.9.e67169.suppl1 Supplementary material 1 Species biodiversity transnational geo-database - IMPRECO Project Data type Checklist File: oo_544906.txt https://binary.pensoft.net/file/544906 Francesco Zangaro, Gabriele Marini, Valeria Specchia, Matteo De Luca, Giovanna Bullo, Jacopo Richard, Nataša Šalaja, Bia Rakar, Bojana Lipej, Jelena Kurtović Mrčelić, Gvido Piasevoli, Nada Zaimi, Djana Bejko, Abdulla Diku, Aliki Karousou, Eleni Hatziyanni, Maurizio Pinna

F8169B79-B3BC-5CF2-A615-1981DDA09A07 10.3897/BDJ.9.e67169.suppl2 Supplementary material 2 Habitats biodiversity transnational geo-database - IMPRECO Project Data type Coverage File: oo_521713.txt https://binary.pensoft.net/file/521713 Francesco Zangaro, Gabriele Marini, Valeria Specchia, Matteo De Luca, Giovanna Bullo, Jacopo Richard, Nataša Šalaja, Bia Rakar, Bojana Lipej, Jelena Kurtović Mrčelić, Gvido Piasevoli, Nada Zaimi, Djana Bejko, Abdulla Diku, Aliki Karousou, Eleni Hatziyanni, Maurizio Pinna
